# Fascin-1, Ezrin and Paxillin Contribute to the Malignant Progression and Are Predictors of Clinical Prognosis in Laryngeal Squamous Cell Carcinoma

**DOI:** 10.1371/journal.pone.0050710

**Published:** 2012-11-27

**Authors:** Wei Gao, Chunming Zhang, Yan Feng, Ganggang Chen, Shuxin Wen, Hui Huangfu, Binquan Wang

**Affiliations:** 1 Department of Otolaryngology, Head & Neck Surgery, No. 1 Hospital, Shanxi Medical University, Taiyuan, Shanxi, China; 2 Key Institute and Laboratory of Otolaryngology affiliated with Shanxi Province, Taiyuan, Shanxi, China; Emory University, United States of America

## Abstract

**Aims:**

Fascin-1, ezrin and paxillin, cytoskeleton-associated proteins, have been implicated in several human cancers, but their role in laryngeal squamous cell carcinoma (LSCC) is unknown. We investigated the association of their expression and clinicopathologic factors and their prognostic value in LSCC.

**Materials and Methods:**

Quantitative RT-PCR and western blot analyses were used to examine mRNA and protein levels in 10 fresh LSCC specimens and 10 corresponding adjacent normal margin (ANM) tissues from patients undergoing surgery in 2012. We used immunohistochemistry to retrospectively study 216 paraffin blocks of LSCC samples from patients (193 men) who had undergone surgery between 2000 and 2006 and had not received special treatment before the diagnosis. Univariate analysis of patient survival involved the Kaplan–Meier method. Multivariate analyses involved the Cox proportional hazards model.

**Results:**

The relative mRNA and protein levels of fascin-1, ezrin and paxillin were significantly greater in LSCC than ANM tissue (*P*<0.05). The high expression of fascin-1, ezrin or paxillin was positively correlated with poor tumor differentiation, cervical lymph node metastasis (N+), and advanced clinical stage (III+IV) (*P*<0.05) but not sex or metastasis. In addition, a high expression of fascin-1 (*P* = 0.007) or ezrin (*P* = 0.047) was associated with advanced tumor stage (T3+T4). The expression of fascin-1 was higher in smokers than non-smokers (*P* = 0.019). A high expression of fascin-1, ezrin or paxillin was associated with poor prognosis.

**Conclusions:**

Fascin-1, ezrin and paxillin may be prognostic of poor outcome with LSCC after surgery. Our study may lead to establishing new molecular therapeutic targets and/or prognostic biomarkers in LSCC.

## Introduction

Head and neck squamous cell carcinoma, the 11th most common cancer among men worldwide [1], [2], is one of the 6 tumors with a high incidence in China [3]. Laryngeal squamous cell carcinoma (LSCC), which originates from the laryngeal epithelium, has the second highest incidence of all head and neck squamous cell carcinomas, especially in the northern area of China, including Shanxi Province [4]. LSCC is a highly invasive and metastatic cancer [5]; this malignant behavior is one of the major reasons for treatment failure, which explains why the survival of LSCC patients has not improved much over years. Identifying some molecular indicators of the malignant behavior can be helpful for early prevention, diagnosis and treatment.

The incremental motility of malignant cells is a critical step in their migration, invasion and metastasis, which is regulated by reorganization of actin cytoskeleton and regulation of focal adhesion [6], [7], [8], [9]. Fascin-1, ezrin and paxillin are essential components of these cellular structures.

Fascin-1 was originally described in the extracts of unfertilized sea urchin eggs as an actin-binding protein of 55 kDa. It is encoded by a gene located on chromosome 7p22 in humans [10]. The factor is responsible for actin bundle rearrangement, promoting tumor cell invasion and metastasis by increasing cell membrane protrusions, changing cell–cell and cell–extracellular matrix adhesion and regulating signal transduction pathways [10], [11], [12]. Fascin-1 overexpression was found associated with unfavorable prognosis in many malignant tumors such as colon cancer [11], gastric cancer [13], oral squamous cell carcinoma [14],esophageal squamous cell carcinoma (ESCC) [15], non-small-cell lung cancer (NSCLC) [16], and breast cancer [17]. Overexpression of fascin-1 was found associated with aggressiveness of LSCC but in a limited number of subjects [18], [19].

**Table 1:** Patient population and clinicopathological variables.

Variable	No. (Percentage)
**Median age (years)**	59.0 (range 18–82)
**Gender**	
Female	23 (10.6%)
Male	193 (89.4%)
**Primary tumor sites**	
Supraglottic	96 (44.4%)
Glottic	116 (53.7%)
Subglottic	4 (1.9%)
**Histologic differentiation**	
Well	84 (38.9%)
Moderate	89 (41.2%)
Poor	43 (19.9%)
**Primary tumor**	
T1	59 (27.3%)
T2	67 (31.0%)
T3	48 (22.2%)
T4	42 (19.4%)
**Cervical lymph node metastasis**	
N0	161 (74.5%)
N1	41 (19.0%)
N2	13 (6.0%)
N3	1 (0.5%)
**Distant metastasis**	
M0	211 (97.7%)
M1	5 (2.3%)
**Clinical stage**	
I	56 (25.9%)
II	48 (22.2%)
III	59 (27.3%)
IV	53 (24.5%)
**Smoking status**	
Yes	132 (61.1%)
No	84 (38.9%)

**Figure 1:**
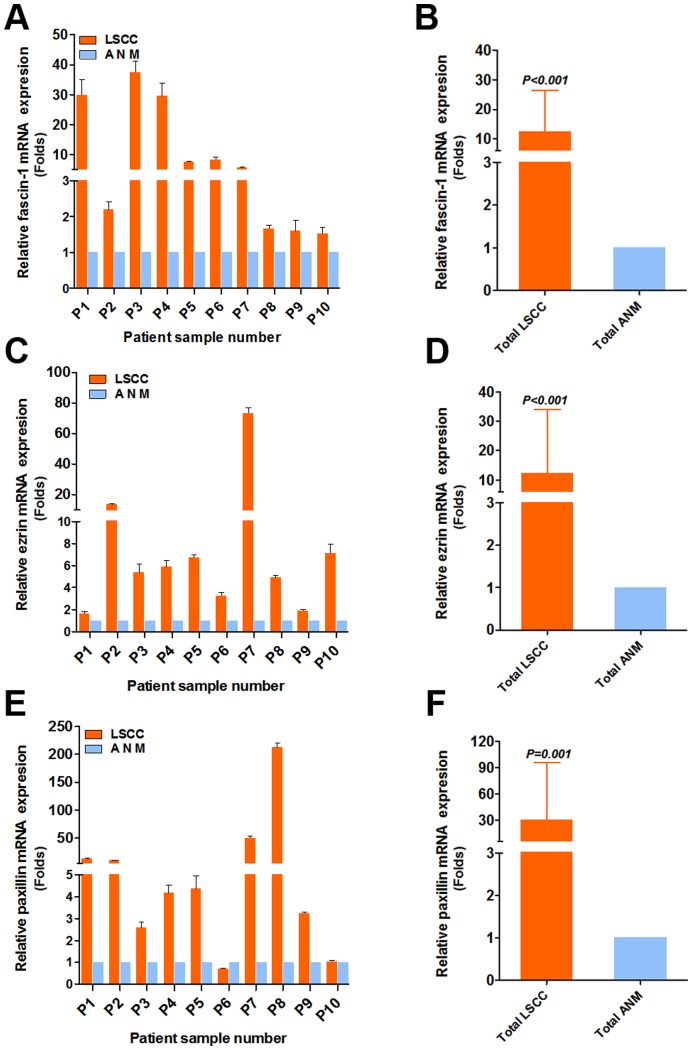
Quantitative RT-PCR analysis of relative mRNA level of fascin-1 (A), ezrin (C) and paxillin (E) in laryngeal squamous cell carcinomas (LSCC) and adjacent normal margin (ANM) tissue by sample pairs. Levels were relative to that of 18s RNA as an internal control. Data are mean±SD from experiments performed in triplicate.

Ezrin is a member of the ezrin-radixin-moesin (ERM) family that links actin to the cell-membrane–associated proteins and plays a role in growth-factor–receptor signaling [20], including cytoskeleton regulation [21] and intercellular adhesion [22]. Overexpression of ezrin is associated with invasion and metastasis in head and neck squamous cell carcinoma (HNSCC) [23], pancreatic carcinomas [24], ovarian cancer [25], breast cancer [26], [27], ESCC [28], osteosarcoma [29] and lung carcinoma [30], [31].

Paxillin, a 68-kDa focal adhesion-associated protein, plays an important role in controlling cell spread and migration. Previous research has demonstrated paxillin at the interface between the plasma membrane and the actin cytoskeleton. It provides a platform for the integration and processing of adhesion and growth factor-related signals that are mainly involved in tumor metastasis and cell proliferation [32], [33]. Paxillin is overexpressed in many carcinomas, including bladder cancer [33], ESCC [34], [35], hepatocellular carcinoma (HCC) [36], NSCLC [37], salivary adenoid cystic carcinoma [38], and floor of mouth carcinoma [39].

**Figure 2:**
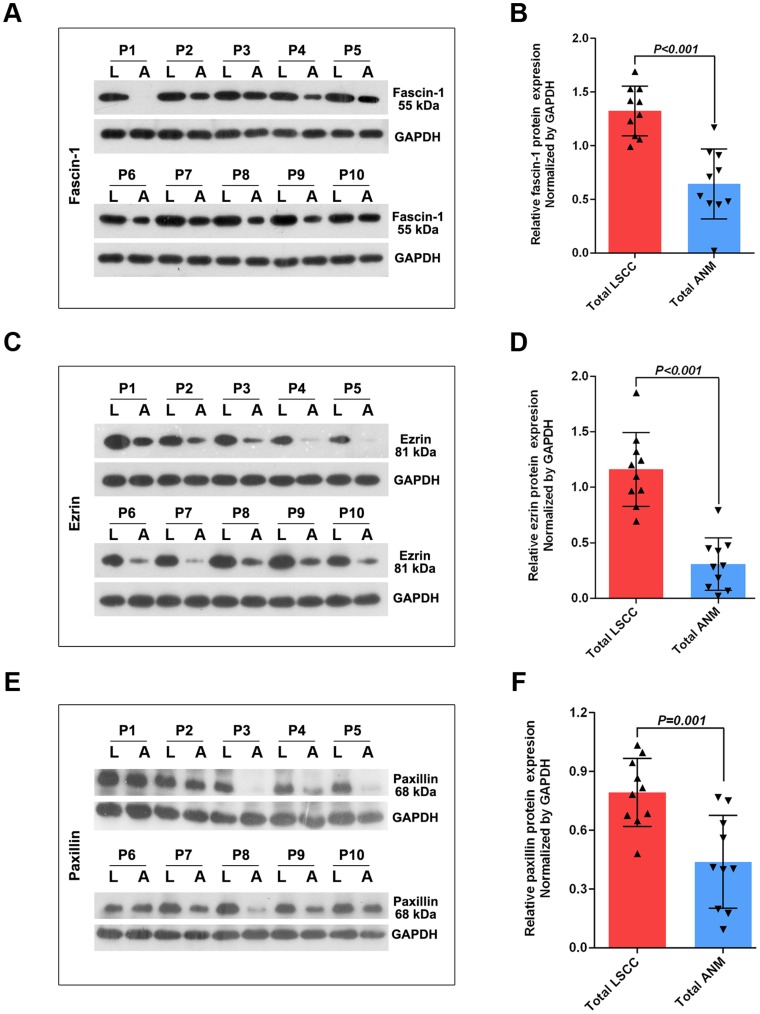
Western blot analysis and quantification of protein levels of fascin-1 (A), ezrin (C) and paxillin (E) in LSCC (L) and ANM (A) tissues (P1-P10). Data are normalized by the loading control GAPDH. Bars are mean±SD fold increase or decrease from experiments performed in triplicate. Arrowheads represent data for each subject.

Thus, fascin-1 is an actin-binding protein that promotes tumor cell invasion and metastasis, ezrin is a cross-linker of the actin cytoskeleton and the plasma membrane and plays a role in growth-factor–receptor signaling, and paxillin is a focal-adhesion–associated protein that plays an important role in controlling cell spread and migration. Thus, these proteins may contribute to the malignant behavior of LSCC. We aimed to detect the expression of these cytoskeleton-associated proteins in human LSCC and to correlate their expression with clinicopathological features and clinical outcomes.

**Figure 3:**
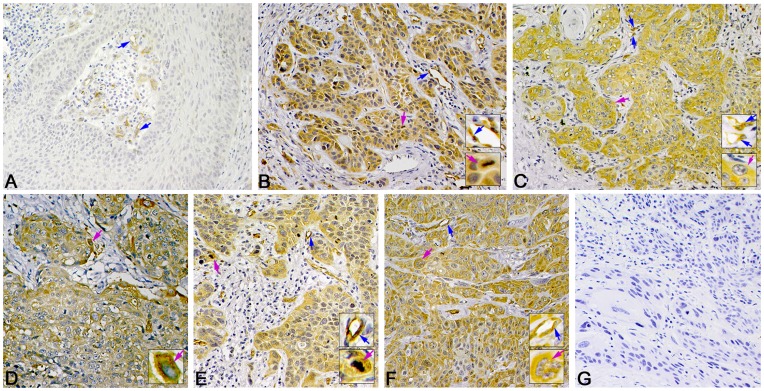
Representative immunohistochemistry staining of fascin-1 expression in LSCC. Positive immunostaining of fascin-1 is in cytoplasm of tumor cells (pink arrows) and in endothelial cells of microvessels (blue arrows). (A) Negative expression of fascin-1 in well-differentiated LSCC. High expression of fascin-1 in (B) well-differentiated, (C) poorly differentiated, (D) supraglottic, (E) glottic, and (F) subglottic LSCC. (G) Negative control staining (phosphate buffered saline [PBS]) of fascin-1. (Magnification 400×, insets 1000×).

**Figure 4:**
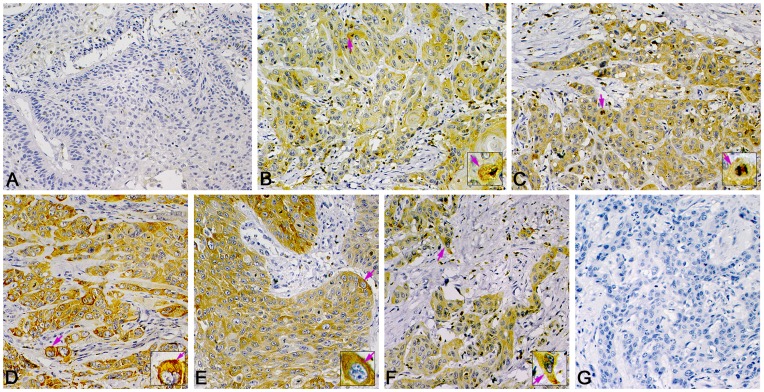
Representative immunohistochemistry staining of ezrin in LSCC. Positive immunostaining can be seen in the cytoplasm of tumor cells (pink arrow). (A) Negative expression of ezrin in well-differentiated LSCC. Little positive immunostaining in the cytoplasm of tumor cells. High expression of ezrin in (B) well-differentiated, (C) poorly differentiated, (D) supraglottic, (E) glottic, (F) and subglottic LSCC. (G) Negative control staining (PBS) of ezrin. (Magnification 400×, insets 1000×).

**Figure 5:**
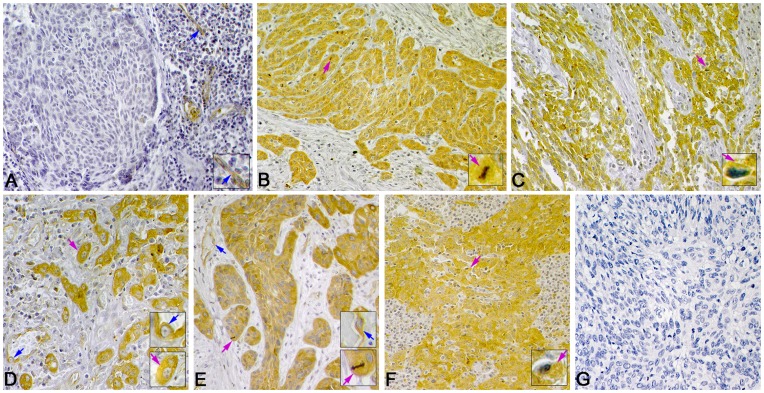
Representative immunohistochemistry staining of paxillin in LSCC. Positive immunostaining of paxillin mainly in the cytoplasm of tumor cells (pink arrows) and in endothelial cells of microvessels (blue arrows). (A) Negative expression of paxiliin in well-differentiated LSCC. (B) High expression of paxillin in well-differentiated, (C) poorly differentiated, (D) supraglottic, (E) glottic, and (F) subglottic LSCC. (G) Negative control staining (PBS) of paxillin. (Magnification 400×, insets 1000×).

**Table 2:** Association of expression of cytoskeleton proteins fascin-1, ezrin and paxillin and clinicopathological variables.

	Fascin-1, no. (%)				Ezrin, no. (%)				Paxillin, no. (%)			
Clinicopathological variables	Negative	Low	High	*P*	Negative	Low	High	*P*	Negative	Low	High	*P*
**Median age**												
<59 years	11(10.3)	30(28.0)	66(61.7)	0.281*	13(12.1)	45(42.1)	49(45.8)	***<0.001*** *	12(11.2)	25(23.4)	70(65.4)	0.532*
≥59 years	7(6.4)	24(22.0)	78(71.6)		8(7.3)	21(19.3)	80(73.4)		10(9.2)	20(18.3)	79(72.5)	
**Gender**												
Female	3(13.0)	4(17.4)	16(69.6)	0.518*	4(17.4)	6(26.1)	13(56.5)	0.412*	4(17.4)	3(13.0)	16(69.6)	0.355**
Male	15(7.8)	50(25.9)	128(66.3)		17(8.8)	60(31.1)	116(60.1)		18(9.3)	42(21.8)	133(68.9)	
**Primary sites**												
Glottic	13(11.2)	42(36.2)	61(52.6)	<0.001**	14(12.1)	38(32.8)	64(55.1)	0.380**	16(13.8)	22(19.0)	78(67.2)	0.310**
Supraglottic	5(5.2)	12(12.5)	79(82.3)		7(7.3)	28(29.2)	61(63.5)		6(6.2)	23(24.0)	67(69.8)	
Subglottic	0(0.0)	0(0.0)	4(100.0)		0(0.0)	0(0.0)	4(100.0)		0(0.0)	0(0.0)	4(100.0)	
**Histologic differentiation**												
Well	11(13.1)	38(45.2)	35(41.7)	<0.001**	15(17.9)	38(45.2)	31(36.9)	<0.001**	16(19.0)	31(36.9)	37(44.1)	<0.001**
Moderate	5(5.6)	12(13.5)	72(80.9)		4(4.5)	20(22.5)	65(73.0)		4(4.5)	8(9.0)	77(86.5)	
Poor	2(4.7)	4(9.3)	37(86.0)		2(4.7)	8(18.6)	33(76.7)		2(4.7)	6(14.1)	35(81.4)	
**Primary tumor**												
T1+ T2	11(8.7)	41(32.6)	74(58.7)	***0.007*** *	14(11.1)	44(34.9)	68(54.0)	0.125*	14(11.1)	30(23.8)	82(65.1)	0.332*
T3+ T4	7(7.8)	13(14.4)	70(77.8)		7(7.8)	22(24.4)	61(67.8)		8(8.9)	15(16.7)	67(74.4)	
**Cervical lymph node metastasis**												
N0	17(10.5)	46(28.6)	98(60.9)	***0.006*** *	20(12.4)	54(33.5)	87(54.1)	***0.007*** *	20(12.4)	38(23.6)	103(64.0)	***0.021*** *
N+	1(1.8)	8(14.6)	46(83.6)		1(1.8)	12(21.8)	42(76.4)		2(3.6)	7(12.7)	46(83.6)	
**Distant metastasis**												
M0	18(8.5)	54(25.6)	139(65.9)	0.566**	21(10.0)	65(30.8)	125(59.2)	0.806**	22(10.4)	45(21.3)	144(68.2)	0.758**
M1	0(0.0)	0(0.0)	5(100.0)		0(0.0)	1(20.0)	4(80.0)		0(0.0)	0(0.0)	5(100.0)	
**Clinical stage**												
I+ II	10(9.6)	36(34.6)	58(55.8)	***0.003*** *	13(12.5)	37(35.6)	54(51.9)	0.071*	14(13.5)	27(26.0)	63(60.6)	***0.035*** *
III+ IV	8(7.1)	18(16.1)	86(76.8)		8(7.1)	29(25.9)	75(67.0)		8(7.1)	18(16.1)	86(76.8)	
**Smoking status**												
No	12(14.3)	23(27.4)	49(58.3)	***0.023*** *	7(8.3)	35(41.7)	42(50.0)	***0.018*** *	9(10.7)	23(27.4)	52(61.9)	0.145*
Yes	6(4.5)	31(23.5)	95(72.0)		14(10.6)	31(23.5)	87(65.9)		13(9.8)	22(16.7)	97(73.5)	

* Pearson chi-square, ** Fisher's Exact Test.

## Materials and Methods

### Ethics Statement

This study was conducted in accordance with the Helsinki declaration. All patients signed a written, informed consent before surgery, acknowledging that they understood their rights and obligations. The study was approved by the Research Ethics Committee at Shanxi Medical University.

### Clinical Samples and Patient Population

Ten LSCC tissues and 10 corresponding adjacent normal margin (ANM) tissues were obtained from patients undergoing surgery at the Department of Otolaryngology Head and Neck Surgery of the First Hospital of Shanxi Medical University in 2012. ANM tissues were isolated from surgical specimens at about 1 to 3 cm from the neoplastic edge. The fresh specimens were divided into 3 parts: 2 parts were frozen by use of liquid nitrogen for quantitative RT-PCR (qRT-PCR) and western blot analyses, and the other part was embedded in paraffin for hematoxylin and eosin staining to ensure the diagnosis of LSCC and ANM.

We examined data for 216 eligible patients (193 males) with LSCC who had detailed clinical records and had not received special treatment before the diagnosis. The patients underwent surgery in our department from 2000 to 2006. Two staff anatomical pathologists gave the diagnosis of laryngeal cancer. Patients did not receive radiotherapy or chemotherapy. Date of laryngeal cancer surgery, date of last follow-up and status at last follow-up (living, lost to follow-up or deceased) were recorded.

Tumor and clinical staging of paraffin-embedded tissues involved the tumor, node, metastasis (TNM) staging system of the Union for International Cancer Control (2010). The histological types of LSCC were determined according to the system of the World Health Organization.

### Purification of Total RNA and qRT-PCR

Total RNA was purified from the frozen cancer tissues by use of a RNAiso Plus reagent kit (TaKaRa); RNA contents were determined 3 times in BioPhotometer plus (Eppendorf) to ensure OD_260_/OD_280_≥1.8 and in 1% agarose gel electrophoresis to ensure integrity. Reverse transcription of total RNA (1 µg) involved use of the Rever Tra Ace qPCR RT Kit (TOYOBO); cDNA was amplified by qPCR with the SYBR® Green Realtime PCR Master Mix (TOYOBO) and the ABI PRISM® 7500 Sequence Detection System (Applied Biosystems). The cycling conditions included a 5-min initial denaturation step at 95°C, then 40 cycles at 94°C for 15 s, 60°C for 15 s, and 72°C for 32 s. The primer sequences were for fascin-1, forward 5′-AGCTGCTACTTTGACATCGA-3′ and reverse 5′-TCATGAGGAAGAGCTCTGAGT-3′ (139 bp); ezrin, forward 5′-AGCTGTGAAGAGACTCTGTTTG-3′, and reverse 5′-CTTAGCTGTGAAGGAGAAAGC-3′ (150 bp); and paxillin, forward 5′- CATATCGCCTGAGTTGCTT-3′, and reverse 5′-CACCTGCTTGTGCAAGAAA-3′ (200 bp). The Ct values were normalised with 18s RNA as an internal control, which was amplified with the primer sequences, forward 5′-CCTGGATACCGCAGCTAGGA-3′, and reverse 5′-GCGGCGCAATACGAATGCCCC-3′. All reactions were performed in triplicate, and the experiment was repeated 3 times.

### Western Blot Analysis

Tissue protein lysates were obtained by use of the Tissue Protein Extraction Reagent and Proteasome Inhibition Mixture (Cwbiotech). Briefly, fresh tissue was rinsed with 4°C phosphate buffered saline (PBS) to remove blood on the surface, placed in liquid nitrogen for 2 to 5 s, then immediately ground into a fine powder. The powder was collected into an EP tube (Axygene) with cold lysis buffer and incubated on ice for 60 min. The lysate was centrifugated at 10000×g, 4°C for 20 min in the Centrifuge 5702R (Eppendorf). Protein concentrations were determined by use of the bicinchoninic acid protein assay kit (Cwbiotech). Protein with 2× loading buffer (0.25 mol/L Tris-Cl, pH 6.8, 10% SDS, 0.5% bromophenyl blue, and 50% glycerol) was boiled for 5 min, loaded into 10% Tris-HCl polyacrylamide gels (80v, 50 min), then electrophoretically transferred to Immobilon-P Transfer Membrane (Millipore Corp.) and blocked with 5% nonfat milk at 4°C overnight and then incubated with primary antibodies for fascin-1 (mMAb IM20, 1∶550, Vector Laboratories), ezrin (mMAb 3C12, 1∶500, Abcam) and paxillin (mMAb 5H11, 1∶700, Thermo Fisher Scientific) at 4°C overnight, then with rabbit anti-mouse IgG H+L (horseradish peroxidase conjugate [HRP], 1∶4000) (Southern Biotech) for 2 hr at room temperature. Immunoreactive bands were detected and developed with use of Immobilon Western chemilum HRP substrate (Millipore Corp.) and X-ray film (Kodak). HRP-conjugated monoclonal mouse glyceraldehyde-3-phosphate dehydrogease (GAPDH) from KangChen Biotech was used as loading control. The same analyses were performed 3 times. Analysis involved use of Quantity One v4.0 (Bio-Rad).

### Antibodies and Immunohistochemistry Staining

Paraffin sections were dewaxed and re-hydrated in ethanol in descending concentrations (100%, 90%, 80%, 70%). For antigen retrieval, samples were processed in an autoclave as follows: fascin-1 for 2′15 with sodium citrate pH 6.0; ezrin, for 2′00′′ with sodium citrate pH 6.0, and paxillin for 2′10′′ with EDTA, pH 9.0. Endogenous peroxidase activity was blocked by immersing tissue sections in 3% H_2_O_2_ in methanol (v/v) at room temperature for 10 min and then washing with PBS. Nonspecific background staining was reduced by incubating sections with normal nonimmune goat serum (Boster Co.) for 15 min at room temperature. The sections were in a moist chamber and incubated overnight at 4°C with primary antibodies for fascin-1 (mMAb IM20, 1∶200; Vector Laboratories), ezrin (mMAb 3C12, 1∶100) and Paxillin (mMAb 5H11, 1∶100; both Thermo Fisher Scientific) and washed 3 times with PBST (containing PBS and 1‰ Tween20) for 5 min each. Sections were incubated with the secondary antibody from the Max VisionTM HRP-Polymer anti-Mouse IHC kit (MaxinBio) for 15 min at room temperature, then washed 3 times for 5 min with PBST. The DAB substrate detection system was used (Vector Laboratories, Inc.). Counterstaining involved hematoxylin, then sections were dehydrated and mounted with coverslips.

### Evaluation and Scoring of Immunohistochemistry

Each immunohistochemically stained sample was independently evaluated by 2 pathologists with agreements,but adjuster when argued (Pathology Department, No. 1 Hospital Affiliated with Shanxi Medical University). The slides were coded and evaluators were not aware of the code, to avoid the observer bias. All images were captured by use of the MShot Digital CCD Transducer Imaging System (Microshot Technology Co.). To determine the expression pattern, at least 10 high-power fields (40×10) and at least 100 cancer cells per field were selected randomly for microscopy.

Squamous lung carcinoma was selected as an appropriate positive control for fascin-1, ezrin and paxillin [16], [31], [37]. Negative controls were obtained by substituting the primary antibody with PBS.

Immunohistochemical staining was assessed semiquantitatively by the proportion of tumor cells with positive staining and the staining intensity showing an unequivocal positive reaction defined as brown signals in the cell cytoplasm or membrane. Staining intensity, distinguished from background noise and squamous lung carcinoma, was scored as 0, no staining (same as negative controls); 1, weak intensity; 2, moderate intensity; 3, strong intensity. Positive staining percentage was scored as 1, ≤10%; 2, 11–50%; 3, 51–80%; and 4, ≥81%. The final assessment was the mean combined score of staining intensity and positive staining percentage, range 1 to 7, calculated 3 times. According to the overall score, specimens were classified into 4 grades: “−”, <10% of cells stained positive regardless of intensity; “1+”, 3 points; “2+”, 4–5 points; and “3+”, 6–7 points. For statistical analyses, specimens were classified by a three-tier semiquantitative scheme: “−”, negative expression; “1+”, low expression; and “2+” and “3+”, high expression [14], [19], [40].

### Statistical Analysis

Statistical analysis involved use of SPSS v19.0 (SPSS Inc., Chicago, IL). For qRT-PCR, the relative mRNA level was analyzed by the 2^−ΔΔCT^ method [41]. The relative mRNA or protein fold change in level between total LSCC and total ANM samples involved the Mann-Whitney or Student *t* test.

The association of immunohistochemical status and clinicopathological variables was assessed by Pearson’s chi-square or Fisher’s exact test and that of fascin-1, ezrin and paxillin immunohistochemical staining and clinical and histopathological variables by Mann-Whitney or Kruskal-Wallis test. The correlation between the expression of the 3 proteins was assessed by Kendall’s tab-b correlation analysis. Overall survival was defined as the interval from the date of surgery to death from laryngeal carcinoma or date of last contact. Survival was analyzed by the Kaplan–Meier method with the Log rank test. Multivariate analyses involved use of Cox proportional hazards models. For all tests, a two-tailed P≤0.05 was considered significant. Data display involved use of GraphPad Prism v6.0 Demo (San Diego, CA).

**Table 3:** Correlation between fascin-1, ezrin and paxillin immunostaining rank and clinicopathological characteristics.

		Mean rank					
Clinicopathological characteristics	No.	Fascin-1	*P*	Ezrin	*P*	Paxillin	*P*
**Median age**							
<59 years	107	102.86	0.113	93.86	***<0.001***	104.70	0.277
≥59 years	109	114.03		122.87		112.23	
**Gender**							
Female	23	10967	0.908	102.04	0.547	106.65	0.854
Male	193	108.36		109.27		108.72	
**Primary sites**							
Glottic	116	93.53	***<0.001*** *	94.68	0.248*	105.60	0.320*
Supraglottic	96	125.09		100.33		110.60	
Subglottic	4	144.50		131.00		142.00	
**Histologic differentiation**							
Well	84	82.04	***<0.001*** *	77.30	**<0.001** *	81.35	***<0.001*** *
Moderate	89	123.57		108.06		127.42	
Poor	43	129.01		119.98		122.40	
**Primary tumor**							
T1+ T2	126	100.50	***0.007***	102.29	***0.047***	104.40	0.161
T3+ T4	90	119.70		117.20		114.23	
**Cervical lymph node metastasis**							
N0	161	101.96	***0.002***	101.78	***0.002***	102.89	***0.006***
N+	55	127.65		128.16		124.91	
**Distant metastasis**							
M0	211	107.65	0.116	107.93	0.318	107.71	0.136
M1	5	144.50		135.50		142.00	
**Clinical stage**							
I+ II	104	97.25	***0.002***	99.69	***0.022***	99.25	***0.010***
III+ IV	112	118.95		116.68		117.09	
**Smoking status**							
No	84	98.11	***0.019***	99.63	0.056	101.46	0.104
Yes	132	115.11		114.15		112.98	

* By Kruskal-Wallis H(K) test; others by Mann-Whitney U test.

**Table 4:** Correlation of fascin-1, ezrin and paxillin protein expression in 216 samples of LSCC.

	Ezrin		Paxillin	
	Kendall's tab-b coefficient	*P* *	Kendall's tab-b coefficient	*P* *
**Fascin-1**	0.398	***<0.001***	0.463	***<0.001***
**Paxillin**	0.372	***<0.001***		

* Correlation is significant at the 0.01 level (2-tailed).

**Figure 6:**
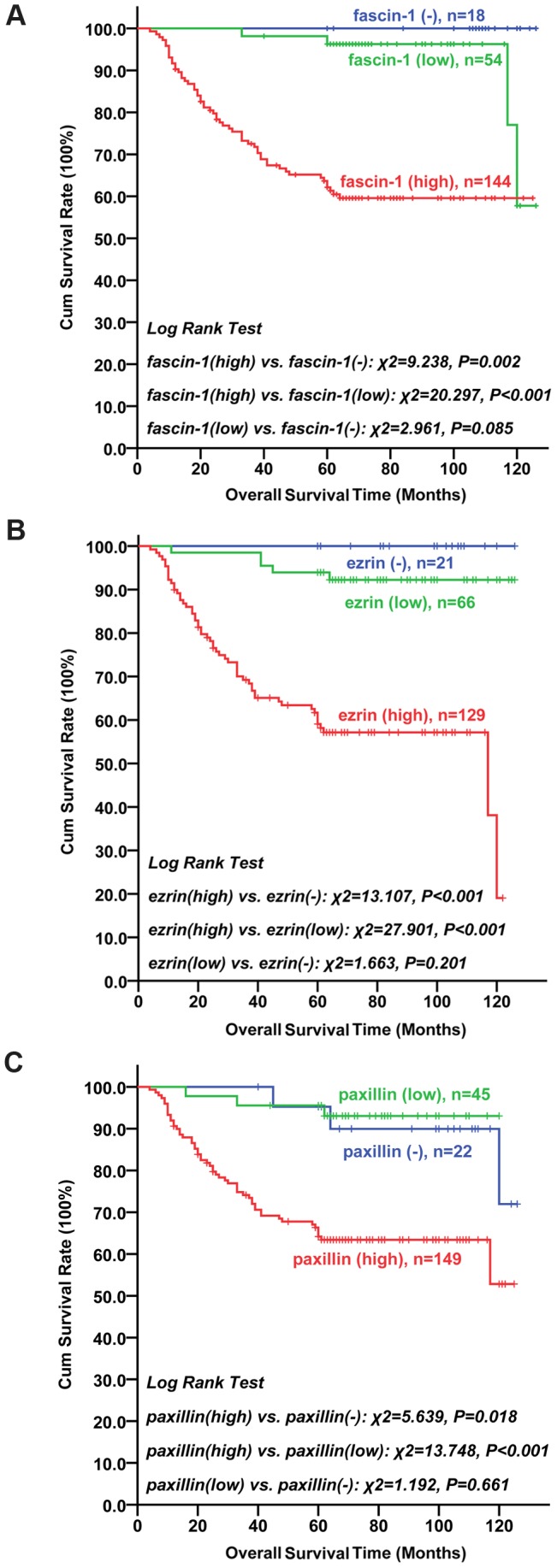
Survival curves by Kaplan-Meier analysis (Log-rank test). LSCC patients with negative or low expression of (A) fascin-1, (B) ezrin or (C) paxillin survived significantly longer than those with high expression.

**Table 5:** Univariate prognostic value of variables for overall survival time with laryngeal squamous cell carcinoma.

		Log Rank Test	
Variables	Categories	Chi-square	*P*
Age	Continuous	160.008	***<0.001***
Gender	Male/female	2.399	0.121
Primary sites	Supraglottic/subglottic/glottic	42.075	***<0.001***
Histologic differentiation	Well/Moderate/Poor	37.506	***<0.001***
Primary Tumor	T1/T2/T3/T4	13.318	***0.004***
Cervical lymph node metastasis	N0/N+	119.032	***<0.001***
Distant Metastasis	M0/M1	58.429	***<0.001***
Smoking	Y/N	17.187	***<0.001***
Fascin-1	Negative/low/high	30.249	***<0.001***
Ezrin	Negative/low/high	39.452	***<0.001***
Paxillin	Negative/low/high	18.512	***<0.001***

**Table 6:** Cox proportional hazards regression analysis (forward stepwise: likelihood ratio).

**Variables**	**Wald statistic**	**Hazard ratio (95% ** ***CI*** **)**	***P***
Age	10.958	1.052(1.020–1.084)	***0.001***
Cervical lymph node metastasis	17.677	3.131(1.839–5.331)	***<0.001***
Distant metastasis	13.728	7.022 (2.504–19.687)	***<0.001***
Histologic differentiation	9.764	1.847(1.257–2.713)	***0.002***
Smoking	7.709	2.671(1.335–5.334)	***0.005***
Fascin-1	5.702	3.607(1.259–10.340)	***0.017***
Ezrin	7.614	3.584(1.447–8.873)	***0.006***

95% CI, 95% confidence interval.

## Results

A summary of study population and clinicopathological variables is in Table 1.

### Expression of Fascin-1, Ezrin and Paxillin in Cancer and Normal Tissue

The mRNA levels of fascin-1, ezrin and paxillin were greater in LSCC than ANM samples, by 12.50±14.03-fold, 12.33±21.66-fold and 30.20±65.70-fold (all P≤0.001), respectively (Figure 1B, 1D, 1F). The protein levels of fascin-1, ezrin and paxillin were greater in LSCC than ANM samples (Figure 2B, D, F; all P≤0.001).

### Immunohistochemistry

Fascin-1, ezrin or paxillin was mainly localized in the cytoplasm of tumor cells (Figures 3, 4 and 5). Immunoreactivity was uniform in the central tumor areas but was increased in the invasion front of the tumor. However, cytoplasmic staining was usually lost in keratinized cells or keratin pearls. Staining for the 3 proteins was positive in the cytoplasm of inflammatory cells (mainly lymphocytes). Staining for fascin-1 and paxillin but not ezrin was found in vascular endothelial cells.

The expression was high for fascin-1 in 66.7% cases (144 of 216), for ezrin in 59.7% cases (129 of 216), and for paxillin was high in 69.0% cases (149 of 216).

### Association of Protein Expression and Clinicopathological Data

The data on the association between the expression ratio distribution of 3 cytoskeleton proteins and clinicopathological characteristics is listed in Table 2 and the correlation is shown in Table 3.

Immunhistochemistry analysis revealed that the expression of ezrin but not fascin-1 or paxillin was higher for patients older than younger than 59 years (mean rank 122.87 vs. 93.86, *P*<0.001; Table 3). For LSCC patients, the expression of fascin-1 was distributed in the subglottis, 100% (4/4); supraglottis, 82.3% (79/96); and glottis, 52.6% (61/116) (*P*<0.001; Table 3). The mean expression was greater in the subglottis than supraglottis and glottis (144.50 vs. 125.09 and 93.53, *P*<0.001), with no association found with ezrin or paxillin.

The expression of the 3 proteins was significantly associated with histological grade (Table 3); it was greater in poorly or moderately differentiated than well-differentiated tumors (*P*<0.001). The expression of fascin-1 was greater in T1+T2 than T3+T4 cancer (mean rank 119.70 vs. 100.50, *P* = 0.007), as was that of ezrin (mean rank 102.29 vs. 117.20, *P* = 0.047) but not paxillin. The expression of the 3 proteins was higher with N+ than N0 cancer (*P*≤0.006) and was higher with stages III+IV than stages I+II cancer (mean rank for fascin-1, 118.95 vs. 97.25, *P* = 0.002; ezrin 116.68 vs. 99.69, *P* = 0.022; paxillin: 117.09 vs. 99.25, *P* = 0.010).

The expression of fascin-1 (*P* = 0.023) and ezrin (*P* = 0.018) was higher for smokers (132 cases) than non-smokers (84 cases). Nevertheless, only fascin-1 expression intensity was higher for smokers than non-smokers (mean rank 115.11 vs. 98.11, *P* = 0.019). The expression of the 3 proteins did not differ by sex or metastasis classification.

### Correlation between Fascin-1, Ezrin and Paxillin Expression in LSCC

The levels of fascin-1, ezrin and paxillin were correlated in LSCC (Table 4). The presence of fascin-1 was significantly correlated with that of ezrin (Kendall's tab-b correlation coefficient = 0.398, *P*<0.001) and paxillin (correlation coefficient = 0.463, *P*<0.001). As well, the presence of ezrin and paxillin was correlated (correlation coefficient = 0.372, *P*<0.001).

### Follow-up and Survival Analysis

The last follow up was carried out in December 2011. The median age of the 216 patients at the time of surgery was 59.0 years (range 18–82 years). The median follow-up was 65 months (range 4–126 months). The data of follow-up was recorded by use of “The otolaryngology follow-up computer program” which was programed by our department. Patients did not undergo radiotherapy, chemotherapy or biotherapy after surgery. Sixteen patients were lost to follow-up. In total, 60 cancer-related deaths occurred, including recurrence *in situ* (37 patients), cervical lymph node metastases (14 patients), stomal recurrence (3 patients), and metastasis to other organs (3 pulmonary, 2 hepatic and 1 bony). The mean survival time of patients with low expression of fascin-1 was 120.14±2.59 months but decreased to 85.86±4.13 months for patients with high expression (*P*<0.001), which significantly differed from patients with negative expression of fascin-1 (*P* = 0.002). Survival time was lower with high than low expression of ezrin (80.27±4.26 vs. 119.40±2.88 months, *P*<0.001). Similarly, survival was lower with high than negative and paxillin expression (88.30±4.02 vs. 117.78±4.83 and 114.30±3.24 months, *P* = 0.018). High expression of fascin-1, ezrin or paxillin was associated with poor prognosis in postoperative LSCC (Figure 6).

### Multivariate Survival Analysis

Significant variables in univariate survival analyses (Table 5) were entered into the Cox proportional hazards regression analysis. Patient age (*P* = 0.001), cervical lymph node metastasis (*P*<0.001), distant metastasis at the time of diagnosis (*P*<0.001), histological grade (*P* = 0.002) and preoperative smoking status (*P* = 0.005) were significant among the prognostic indicators of LSCC survival. Notably, fascin-1 or ezrin expression was a significantly independent predictor of LSCC survival (Table 6). The hazard ratio for fascin-1 expression was 3.607 (95% confidence interval 1.259–10.340, *P* = 0.017) and for ezrin 3.584 (1.447–8.873, *P* = 0.006). Then, paxillin expression was not an independent predictor of survival time.

## Discussion

Our results demonstrate significantly greater expression of fascin-1, ezrin and paxillin in LSCC than ANM tissues, which agrees with observations in ESCC [15], [34], gastric cancer [42], and NSCLC [31], [37]. Fascin-1, ezrin or paxillin might promote the malignant progression of LSCC; therefore, we investigated whether fascin-1, ezrin or paxillin levels could be potential molecular markers to determine LSCC prognosis. We found that high expression of fascin-1, ezrin and paxillin was associated with poor prognosis.

Fascin-1, an actin cross-linking protein, is found in the core actin bundles of cell-surface spikes and projections, which are implicated in cell motility. Goncharuk et al. [43] proposed that fascin-1 may play a role in squamous epithelium malignancy. In addition, overexpression of fascin-1 may be a useful prognostic marker in LSCC to improve the early identification of patients with a potentially highly aggressive clinical course [44]. Likewise, we observed a high protein level of fascin-1 in 66.7% of LSCC patients. We found a positive correlation between high expression of fascin-1 and advanced tumor stage (T3+T4), poor cancer differentiation, N+ cancer, and advanced clinical stage (III+IV). This observation was in line with the work of Durmaz et al. [18] and Zou et al. [19] for LSCC and other researchers for a number of other malignant neoplasms [12], [14].

In the present study, the expression of fascin-1 was greater in supraglottic than glottic LSCC. Supraglottic LSCC represented 33 cases (60.0%) of cervical lymph node metastasis, which decreased to 20 cases (36.4%) with glottic LSCC. A rich lymphatic drainage is one salient feature of laryngeal anatomy in the supraglottic region, where cervical lymph node metastasis often occurs in LSCC [5]. The different expression patterns of fascin-1 in primary sites of LSCC may be due to differential incidences of cervical lymph node metastasis.

For LSCC patients in this study, fascin-1 expression status was a significant independent predictor of survival time after surgery, which was similar to reports by Zou et al. [19] showing high fascin-1 expression correlated with poor disease-free survival (DFS) and an independent predictor of DFS in LSCC. These results were not observed by Durmaz et al. [18]. In other malignant neoplasms, including gastric cancer [13], oral squamous cell carcinoma [14], ESCC [15], NSCLC [16], and breast carcinoma [17], high expression of fascin-1 was correlated with poor patient prognosis and/or decreased DFS, which supports our findings. Jawhari et al. [11] indicated a possible role for fascin-1 in the development of colonic carcinoma through augmented cell motility and enhanced metastatic potential with downregulation of hsa-mir-145/143 [45]. The *in vitro* interference in breast cancer cell lines demonstrated a central role for fascin-1 in regulating the cell morphologic, migration and invasion potential by modulating several metastasis-associated genes. Fascin-1 could downregulate the expression and nuclear translocation of key metastasis suppressor proteins such as breast cancer metastasis suppressor-1 and upregulate NF-kappa B activity. Importantly, fascin-1 upregulated other proteins that are critical for the execution of metastasis, such as urokinase-type plasminogen activator and matrix metalloproteases 2 and 9 [17]. These might be potential or similar mechanisms that could explain why fascin-1 overexpression in the cytoplasm of LSCC cells leads to a more clinically aggressive course.

Interestingly, the expression of fascin-1 was higher in smokers than non-smokers. Smoking is an important etiological factor of LSCC predisposition [46]. Cotinine, a main nicotine metabolite, causes cytoskeletal alterations, which influences cell size and shape and induces a mitotic catastrophe phenotype in NSCLC cells [47]. Fascin-1 and smoking may be cofactors in the development of LSCC, which warrants further research.

Ezrin, a member of the ERM family of proteins, is a key signaling molecule that regulates cell survival, adhesion, migration and invasion. It also plays a role in binding plasma membrane proteins with the actin cytoskeleton, thereby providing an intracellular scaffold for the formation of specialized membrane domains that facilitate signal transduction through a number of growth factor receptors and adhesion molecules [48]. Ezrin is associated with malignant progression and metastasis in a variety of human neoplasias [25], [26], [28], [30], [23]. We found 59.7% of LSCC sections with high ezrin expression, which was similar to findings by Xie et al. [28] for ESCC, Wang et al. [49] for salivary gland adenoid cystic carcinoma, and reports of HNSCC [23]. Zhang et al. [31] found positive expression of ezrin common in stage III NSCLC with lymph node metastasis. These data corroborated the present results, which confirm that high expression of ezrin is related to advanced clinical stage (III+IV) and cervical lymph node metastasis. Meanwhile, Wang et al. indicated that increased ezrin expression was significantly correlated with high lymph nodal metastatic rate in nasopharyngeal cancer [50]. An analysis of hepatitis B-related hepatocellular carcinoma showed that ezrin overexpression was independently associated with poor differentiation and invasion [51], which is consistent with our results. Akisawa et al. [24] investigated the expression of ezrin in 16 pancreatic adenocarcinoma cell lines with different metastatic potential. Two lines, S2-CP9 and S2-VP10, with high metastatic potential, showed significantly higher mRNA and protein levels of ezrin than did other cell lines. Ezrin upregulation was confirmed and considered an important regulator in lung cancer bone metastasis, which was induced by transforming growth factor β [30]. These results suggest a strong correlation between high ezrin expression in LSCC and metastatic potential. Furthermore, we found a high expression of ezrin associated with advanced T stage (T3+T4) and age ≥59 years. These data suggest a key role of ezrin in late LSCC progression and metastasis, which needs further investigation. A high expression of ezrin was related to poor outcome, which supported findings in HNSCC [23], ESCC [28], NSCLC [31], salivary gland adenoid cystic carcinoma [49], nasopharyngeal cancer [50], and endometrioid carcinoma [52]. We found that ezrin expression was an independent prognostic factor of LSCC. Although this was not observed in HNSCC [23], it remained a predictor in other multivariate analyses [28], [31], [49], [50], [52].

Paxillin is an adapter for scaffold protein regulation, signaling and focal adhesion assembly, with the primary function being providing multiple docking sites on the plasma membrane for an array of signaling and structural proteins [53], and controlling the dynamic changes during cell adhesion and cytoskeletal reorganization [54]. It has been linked to many malignancies [33], [34], [35], [36], [37], [38], [39] except LSCC. Paxillin was highly expressed in 69.0% of our cases, which was similar to the proportion found for HCC [36] and urothelial bladder tumor [55]. High expression of paxillin was positively correlated with poor tumor differentiation, cervical lymph node metastasis and advanced clinical stage (III+IV). In HCC, positive paxillin expression was associated with low differentiation and extra-hepatic metastasis [36]. Upregulated paxillin mRNA and protein expression was prevalent in stage III NSCLC [37]. These findings agree with ours for LSCC. However, Li et al. [35] suggested that positive paxillin expression in ESCC, although significantly higher than normal squamous tissue, had no significant relation to clinicopathological variables and in predicting ESCC prognosis. Overall, we observed a correlation between paxillin overexpression and LSCC aggressiveness. High paxillin expression was a predictor of poor survival and relapse-free survival but also an independent predictor of NSCLC [56]. We found that high expression of paxillin indicated poor postoperative LSCC survival. However, paxillin was not an independent predictor of LSCC, which agrees with observations in HCC [36]. Positive ezrin and paxillin protein expression was seen in 99% and 93.7%, respectively, of urothelial tumors; downregulation of ezrin and paxillin in urothelial bladder tumors was associated with aggressive tumor features and invasiveness [55]. Different tumor origin, extracellular environments and engagement of different signaling partners may account for these discrepant results. Nonetheless, the mechanism of paxillin in LSCC is still not well understood. Two studies of HNSCC cell lines (UMSCC-1 and SCC 25) indicated that paxillin overexpression contributed to increasing adhesion ability [57] and that paxillin downregulation played a role in suppressing tumor cell proliferation and angiogenesis [39]. Enhanced Ki-67 protein, a cellular marker for proliferation, increased the growth and proliferation of lung cancer cell H522 *in vivo* with overexpression of paxillin [58]. Increased mutant A127T paxillin expression conferred cell growth, oncogenic transformation, and tumor growth and invasion in a nude mouse model. Moreover, a recent report indicated that paxillin upregulation in response to miR-218 reduction enhanced tumor growth and metastasis in lung cancer [37]. Therefore, paxillin may play an essential role in LSCC, acting as an oncogene and driving the LSCC malignant progression.

Cytoskeleton-associated proteins regulate polarity, differentiation, proliferation, migration and invasion of neoplastic cells by their intimate association with the actin cytoskeletal network, a complex mechanism [9]. Their interactions could contribute to the signal transduction, which is involved in the transforming growth factor β, β-catenin-TCF, MARK, and Twist pathways [9], [12], [28], [33], [30], between cellular surface projections (microvilli and membrane ruffle) and cytoplasmic microfilaments, which might be the structural elements responsible for the migratory invasive properties of LSCC [59]. Fascin-1, ezrin and paxillin are regulated by microRNAs and take part in the modulation of malignant progression in carcinoma [29], [37], [45]. Our study demonstrated a positive correlation among the protein levels of fascin-1, ezrin and paxillin, so they may have a synergistic effect during LSCC invasion. Further studies are needed to understand the mechanism of regulation among these proteins, as well as the involvement of growth factors, transcription factors and microRNAs in LSCC.

In summary, we evaluated the expression patterns of the cytoskeleton-associated proteins fascin-1, ezrin and paxillin in tissue and paraffin sections of LSCC. The 3 proteins may be biomarkers for the LSCC malignant process and effective prognostic markers for poor outcome of patients after surgery. Notably, fascin-1 and ezrin may be independent prognostic factors for LSCC after surgery. This study may provide promising new molecular therapeutic targets, a novel strategy of individual therapeutic or useful biomarkers for prognosis of LSCC. However further studies, such as use of short hairpin RNA to downregulate the expression of the factors, either individually or in combination, in human LSCC cell lines are needed. As well, study is needed to determine any changes in cell behavior (proliferation, cell cycle, migration, invasion, metastasis) *in vitro* and *in vivo*, to confirm the findings.
